# The HLS_19_-COM-P, a New Instrument for Measuring Communicative Health Literacy in Interaction with Physicians: Development and Validation in Nine European Countries

**DOI:** 10.3390/ijerph191811592

**Published:** 2022-09-14

**Authors:** Hanne Søberg Finbråten, Peter Nowak, Robert Griebler, Éva Bíró, Mitja Vrdelja, Rana Charafeddine, Lennert Griese, Henrik Bøggild, Doris Schaeffer, Thomas Link, Zdenek Kucera, Julien Mancini, Jürgen M. Pelikan

**Affiliations:** 1 Department of Health and Nursing Sciences, Faculty of Social and Health Sciences, Inland Norway University of Applied Sciences, P.O. Box 400, 2418 Elverum, Norway; 2 Competence Centre for Health Promotion and Health System, Austrian National Public Health Institute, A-1010 Vienna, Austria; 3 Department of Public Health and Epidemiology, Faculty of Medicine, University of Debrecen, H-4028 Debrecen, Hungary; 4 Communication Unit, National Institute of Public Health, Trubarjeva 2, 1000 Ljubljana, Slovenia; 5 Department of Public Health and Epidemiology, Sciensano, 1050 Brussels, Belgium; 6 Interdisciplinary Centre for Health Literacy Research [ICHL], School of Public Health, Bielefeld University, 33615 Bielefeld, Germany; 7 Public Health and Epidemiology, Department of Health Science and Technology, Aalborg University, 9220 Aalborg, Denmark; 8 Department of Quality Measurement and Patient Survey, Austrian National Public Health Institute, A-1010 Vienna, Austria; 9 Czech Health Literacy Institute, Sokolská 490/31, 120 00 Prague, Czech Republic; 10 Aix Marseille University, APHM INSERM, IRD, ISSPAM, SESSTIM, Cancer, Biomedicine & Society Group, Ligue 2019 Accredited Team, F-13009 Marseille, France; 11 WHO-CC Health Promotion in Hospitals and Health Care, Austrian National Public Health Institute, A-1010 Vienna, Austria

**Keywords:** Calgary-Cambridge Guide framework (C-CG), communicative health literacy, confirmatory factor analysis, data collection modes, HLS_19_, measurement, physician–patient communication, Rasch analysis

## Abstract

Background: Sufficient communicative health literacy (COM-HL) is important for patients actively participating in dialogue with physicians, expressing their needs and desires for treatment, and asking clarifying questions. There is a lack of instruments combining communication and HL proficiency. Hence, the aim was to establish an instrument with sufficient psychometric properties for measuring COM-HL. Methods: The HLS_19_-COM-P instrument was developed based on a conceptual framework integrating HL with central communicative tasks. Data were collected using different data collection modes in nine countries from December 2019 to January 2021 (*n* = 18,674). Psychometric properties were assessed using Rasch analysis and confirmatory factor analysis. Cronbach’s alpha and Person separation index were considered for reliability. Results: The 11-item version (HLS_19_-COM-P-Q11) and its short version of six items (HLS_19_-COM-P-Q6) fit sufficiently the unidimensional partial credit Rasch model, obtained acceptable goodness-of-fit indices and high reliability. Two items tend to under-discriminate. Few items displayed differential item functioning (DIF) across person factors, and there was no consistent pattern in DIF across countries. All items had ordered response categories. Conclusions: The HLS_19_-COM-P instrument was well accepted in nine countries, in different data collection modes, and could be used to measure COM-HL.

## 1. Introduction

In recent decades, research has highlighted the importance of health literacy (HL) as a relevant determinant of health. Among others, low HL is found to be associated with poorer health [1,2,3], unhealthier behaviour and a higher number of visits to healthcare services [3,4,5,6]. There are relatively high proportions of people with low HL, and there is also a social gradient for HL [3,5].

By communicating in a “health literate” way addressing patients’ needs, health professionals can play a critical role in taking the patients’ HL into account and strengthening their HL. Adapting health communication to the patients’ HL might also enable them to play an active role as co-producer in healthcare and be able to make sound health decisions.

The quality of communication between patients and health professionals is one of the patients’ main concerns and the most important reason for (dis-)satisfaction with healthcare [7,8,9]. It has an important impact on trust in healthcare providers and the healthcare system. Successful communication in healthcare is associated with a wide range of improved healthcare outcomes and does also contribute to the workplace satisfaction of health professionals [10,11,12]. This indicates the importance of communication proficiency and responsibility for good communication skills on the side of health professionals, who must be regularly trained in this regard.

Patients (the term is used here as a synonym also for healthcare users, clients, citizens, individuals, and people) strongly rely on personal communication with health professionals [13] to understand and manage their health issues. Thus, health(care) communication and patient participation in healthcare have been recognized as a decisive part of HL, as well as a critical determinant of successful disease management and health outcomes [14,15]. However, patients with low HL tend to report poorer communication skills in dialogue with health professionals than those with higher HL [16,17]. Furthermore, patients with lower literacy are less likely to ask questions to health professionals [18]. Hence, sufficient communicative skills of health professionals are especially necessary to actively involve patients with low HL in the dialogue, so they can use health information to manage their health situation properly. Information about patients’ communicative HL (COM-HL) is, thus, needed to better adapt health communication accordingly. In addition, personal communication seems to be an important support for patients to navigate the healthcare system. Smith et al. [19] claim that interactive HL, or COM-HL, comprises the skills to interact with both the healthcare system and the providers. Communication is also related to the theoretical framework and definition of navigational HL [20]. Hence, COM-HL is seen as very relevant for “navigational HL” [20,21].

Nutbeam [22,23,24] stresses the importance of interactive processes by defining communicative/interactive HL as one of three relevant types of HL and linking it to greater autonomy and personal empowerment. Expanding Nutbeam’s conceptualization of communicative/interactive HL by integrating the HL framework of the European Health Literacy Survey (HLS-EU) Consortium [25,26], the following definition of COM-HL in healthcare is proposed: “Communicative health literacy refers to patients’ communicative and social skills that enable them to actively engage in face-to-face encounters with healthcare professionals, to give and seek information, derive meaning from it, and apply this information in decision making and in co-producing their health care” [27] (p. 235).

So far, there is no instrument measuring COM-HL as an independent construct [28]. Nutbeam [23] highlights the need for a specific measurement tool for these “oral literacy and social skills”. Based on Nutbeam’s concept, Ishikawa et al. [29] developed the “Functional, Communicative and Critical HL” scale, where the dimension intending to measure COM-HL consists of five items. However, these items are more about processing health information in general and do not comprise patients’ ability to interact with healthcare professionals. On the other hand, Chinn & McCarthy [30] suggested three items on COM-HL in relation to doctor or nurse interaction. However, these items were a part of a scale intending to also measure functional and critical HL. Hence, the COM-HL items were not considered or tested as a stand-alone scale. The 47-item version of the European HL-Survey Questionnaire (HLS-EU-Q47) [26] also includes a few items concerning COM-HL but without systematic reference to the broader research on healthcare communication. O’Hara et al. [31] published the concept of the “Conversational Health Literacy Assessment Tool (CHAT)” to provide a short actionable survey tool for the clinical context to assess patients’ ability to interact with health professionals, but the 10 items mainly focus on general health information-seeking behaviour and health promotion activities. Only one item focuses on interactive behaviour.

In summary, previous instruments meant to measure COM-HL were either developed to measure only certain communicative tasks or outcomes or to capture only certain aspects of healthcare communication. As far as we know, no previous instrument integrated systematic findings from communication research and HL research into one instrument. We would also like to consider COM-HL as a relational proficiency. Hence, there is a need for a new instrument that covers the COM-HL skills necessary for actively participating in health communication with healthcare professionals, especially physicians.

Based on this background, this article aims to establish an international instrument with sufficient psychometric properties, intending to measure communicative health literacy in patient–physician communication.

## 2. Materials and Methods

### 2.1. Development of the Instrument for Measuring Communicative Health Literacy in Patient–Physician Communication

**Figure 1:**
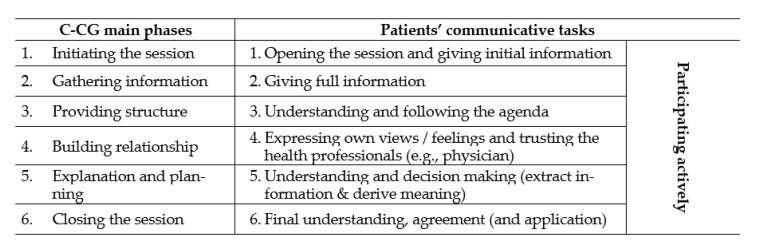
Overview of the main communicative practices of health professionals of the Calgary-Cambridge Guide to the Medical Interview (C-CG) and main communicative tasks of patients, constituting the Conceptual Framework for Communicative Health Literacy.

The new COM-HL with physicians in healthcare instrument, HLS_19_-COM-P, was developed in the context of the Health Literacy Population Survey Project 2019–2021 (HLS_19_), a European HL survey within 17 participating countries from the WHO-Europe region [3]. The HLS_19_-COM-P is based on a comprehensive theoretical framework that integrates Nutbeam’s [22] idea of COM-HL, the basic competencies of information processing according to the HL framework of the HLS-EU Consortium [25,26], and the main communicative tasks of the Calgary-Cambridge Guide framework (C-CG) [32]. The C-CG framework has been developed over the last 25 years and integrates the results of different research traditions to serve as a guide to teach health professionals in patient-centred communication skills. This framework is also used as a framework for assessments of communicative skills of health professionals [33]. The C-CG describes 56 single communicative practices of a health professional in six main phases of a routine interaction in healthcare. Within these six main phases, the C-CG identifies the communicative tasks of patients that need to be considered in the conceptual framework for COM-HL (see Figure 1).

**Figure 2:**
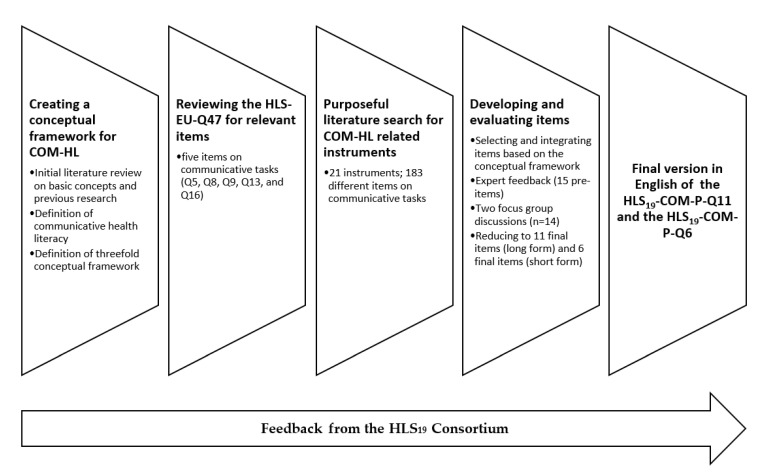
Steps in the development of the instrument to measure communicative health literacy.

Based on our conceptual framework and definition of COM-HL (see above), the HLS_19_-COM-P was developed in a multistage process by a working group of representatives from HLS_19_ countries interested in COM-HL (see Figure 2). After creating the conceptual framework in the first step, the HLS-EU-Q47 [26,34] instrument for measuring general HL was reviewed for suitable items in the second step. Five items on communication in healthcare were identified (Q5, Q8, Q9, Q13 and Q16), but, according to the C-CG, these items only measure aspects of two main phases of interactions between patients and health professionals (explanation and planning; closing the session). In particular, key patient communication tasks were not captured by the HLS-EU-Q47, e.g., presenting their concerns and preferences, and asking questions. Therefore, as a third step, a targeted literature search in English or German language was conducted to identify existing instruments and possible items for measuring COM-HL. In addition to the HLS-EU-Q47, a total of 20 different instruments were found. Since none of these instruments covers all relevant aspects of the underlying conceptual framework and definition of COM-HL, the working group decided to develop a set of items inspired by these instruments. Hence, in the fourth step, the most relevant items were selected from the pool of 183 items and tested in an expert panel and in two focus group interviews. The aim was to identify at least one item per C-CG main phase and the related main communicative task (see Figure 1 and Table 1) to capture the main HL-related challenges in healthcare communication. A preliminary set of 15 items (including 5 items from the HLS-EU-Q47) was selected, which was adapted to the question format of the HLS_19_-HL instruments. In accordance with the HLS_19_ methodology [3], the items are formulated as direct questions (see Table 1) and are rated using a four-point Likert response scale: very easy (4), easy (3), difficult (2), very difficult (1). The comprehensibility and importance of the individual items were assessed in two focus groups involving potential survey participants in Austria (using a German preliminary version of the instrument). Of the focus group participants (*n* = 14), four were men, they were aged 18–54, four had a university degree, eight had high school graduation, and two participants had compulsory school as their highest completed education. Three of the participants were chronically ill. In general, the 15 items were well received and understood. However, the focus group interviews revealed that the term “health professional” was not well accepted by participants because their experiences varied by type of health professional. The term was, therefore, perceived as too vague, making it difficult to form opinions and respond to the items. These general insights were included in creating the original English version. In addition, the set of items and item wording were discussed in several feedback loops within the working group, with the HLS_19_ International Coordination Center, and the HLS_19_ Consortium to ensure transferability to different national contexts. The discussion in the working group indicated that the status of different health professions varies widely in the participating countries, while the status of physicians seems to be quite similar and comparable. Based on these considerations the COM-HL instrument focuses exclusively on physician–patient communication. To create an independent instrument for measuring COM-HL, the five items from the HLS-EU-Q47 were excluded, constituting a set of 11 items (HLS_19_-COM-P-Q11) reflecting the conceptual framework. The HLS_19_-COM-P instrument measures all six main communicative phases of physician–patient interactions according to the C-CG and can be used to analyze the dimensions of COM-HL in accordance with Nutbeam [22] and the basic competencies of information processing according to the conceptual model of HL developed by the HLS-EU Consortium [25,26].

In addition, a 6-item short form (HLS_19_-COM-P-Q6) (see Table 1) was suggested. The short form was proposed based on content considerations, and the shorter length allowed more countries to include HLS_19_-COM-P in their national survey. The short form might also be included more easily in future studies with patients.

### 2.2. Translation Process

The HLS_19_-COM-P instrument was developed in English and translated into eight languages. In six countries (Austria [AT], Belgium [BE] (Dutch translation), Germany [DE], Denmark [DK], Hungary [HU] and Slovenia [SI]), the translation process followed a two-step procedure: first, two forward translations were prepared, one by the National Study Centre (NSC) and one by the data collection agency (DCA), and, second, a comparison of the two translations was carried out by the NSC, with the most appropriate translation being selected in consensus with the DCA in case of differences. The AT and DE versions were also aligned. In three countries (BE (French translation), Bulgaria (BG) and Czech Republic (CZ)), only one forward translation was performed. Back-translation was conducted in CZ and SI. In France [FR], the BE French translation was used with very minor adaptations.

### 2.3. Data Collection

The HLS_19_-COM-P instrument was included as an optional package in the HLS_19_ survey [3]. Countries that chose this optional package could either use the 11-item version (HLS_19_-COM-P-Q11) or the 6-item short version (HLS_19_-COM-P-Q6). Data on COM-HL were collected in nine countries (AT, BE, BG, CZ, DE, DK, FR, HU, and SI). The 11-item version was applied in three of these (AT, DE, and SI). Data were collected using different modes of data collection (Table 2) from December 2019 to June 2021 based on multi-stage random sampling or quota sampling procedures in most countries. A mix of survey methods was used in three countries (BG, CZ, and SI). Except for DE, all surveys took place during the COVID-19 pandemic, which had an impact on possible data collection modes. The sample size in the countries varied from 865 (BG) to 3602 (DK) respondents and data on COM-HL were collected from a total of 18,674 respondents.

### 2.4. Analyses

At the overall level, the psychometric properties of the HLS_19_-COM-P-Q11 and its short version HLS_19_-COM-P-Q6 were assessed using Rasch analysis and by using one-factorial confirmatory factor analysis (CFA). CFA provides detailed information about the overall fit of the model [35] but has some shortcomings (e.g., the results are sample- and scale-dependent and the standard error of measurement is constant) [36,37]. Rasch models are considered as parsimonious models meeting the requirements of fundamental measurement [38]. Rasch analysis also provides detailed information about the items. Hence, Rasch analysis was performed at both overall and item levels [39]. As mentioned above, the HLS_19_-COM-P items are interpreted as ordinal scaled. In the International Report on the Methodology, Results, and Recommendations of the European Health Literacy Population Survey 2019–2021 (HLS_19_) of M-POHL [3], the analyses were based on dichotomous data. In this article, the assessment of psychometric properties is conducted on both the polytomous version of the COM-HL items (very difficult—difficult—easy—very easy) and on a dichotomized version of the COM-HL items (very easy/easy versus difficult/very difficult) to explore which one might be preferable. The internal consistency and reliability were assessed using Cronbach’s alpha and the Person Separation Index (PSI). If conclusions are to be drawn at the individual or group level, the indexes are recommended to exceed 0.85 or 0.65, respectively [40]. Omega for categorical data was used as an index for composite reliability [41]. In addition, the average variance extracted (AVE) was evaluated. An AVE value of ≥0.5 could be considered as acceptable [42]. The analyses were conducted for each country and separately for different modes of data collection if used within a country.

In terms of Rasch analysis, data were tested against the partial-credit parameterization [43] of the unidimensional Rasch model [44]. Analyses at the overall level included data-model fit, targeting (mean person location), and dimensionality [39]. Chi-square statistics were applied to assess the data-model fit. The targeting of the HLS_19_-COM-P-Q11 and the HLS_19_-COM-P-Q6 was assessed by comparing the item and person location distributions on the same metric. An instrument could be deemed as well-targeted if the mean person location values are around zero [45]. Graphical displays for targeting were also inspected. Dimensionality was assessed using the combined procedure of principal component analysis (PCA) of residuals and paired *t*-tests [46,47,48]. Based on the PCA of residuals, two subsets of items were made, and paired *t*-tests were used to examine whether the subsets provided significantly different person location estimates. A scale could be considered sufficiently unidimensional if the proportion of individuals with significantly different person location estimates on the pair of compared subscales does not exceed 5% (or if the lower bound of the binomial 95% confidence interval (CI) does not exceed 5%) [46,48].

CFA was performed for a one-factor model of the HLS_19_-COM-P-Q11 and the HLS_19_-COM-P-Q6 using a WLSMV estimator with diagonally weighted least squares [49,50,51]. The following goodness-of-fit (GOF) indices were considered: standardized root mean square residual (SRMR), root mean square error of approximation (RMSEA), comparative fit index (CFI), Tucker–Lewis index (TLI), goodness-of-fit index (GFI) and adjusted goodness-of-fit index (AGFI). Schumacker and Lomax [52] recommend SRMR < 0.05, RMSEA ≤ 0.05–0.08, CFI, TLI, GFI and AGFI values close to 0.90 or 0.95, whereas Hu and Bentler [53] claim that SRMR values close to or below 0.08 indicate sufficient overall fit. An overview of GOF indices with reference values could also be found in Table S1: Fit indices considered in confirmatory factor analysis.

Rasch analyses at a finer level included assessing item fit, response dependence, ordering of response categories and differential item functioning (DIF) [39]. Chi-square probability values above a Bonferroni-adjusted *p*-value of 5% and fit residuals within the range of ±2.5 indicate adequate item fit [45]. In addition, the mean square residual fit statistic (MNSQ) infit was used to assess item fit. For measuring COM-HL at the population level, infit between 0.7 and 1.3 was considered as sufficient [54]. A residual correlation of <0.3 was applied as an indicator of response dependency. In addition, residual correlations were assessed relative to each other [55]. For ordinal data, the threshold ordering was inspected both statistically and graphically to examine whether the response categories could be considered working as intended. A key requirement of measurement is that items measure invariantly across levels of different person factors, such as gender, age, and education. Lack of invariance in measurement across person factors is called differential item functioning (DIF) [56,57]. Uniform DIF means that there are consistent systematic differences in responses across person factor levels, whereas nonuniform DIF is present if the DIF varies along the latent trait, i.e., the persons factor interacts with the latent trait [56]. Items were inspected for DIF across different levels of person factors (gender, age, educational level, status of employment, ability to pay bills, self-perceived level in society and self-reported general health status), both statistically using two-way analysis of variance of standardized residuals and graphically by inspecting item characteristic curves [58]. Statistical significance was assumed at a Bonferroni-adjusted *p*-value ≤ 5%. An overview of the tests performed with reference values is also found in Table S2: Analyses and tests with reference values considered in Rasch analyses.

Since chi-square statistics are sensitive to sample size, there is a risk of drawing false conclusions due to large sample sizes [59]. Therefore, the amend sample size function in the software RUMM2030 was used to draw a random sub-sample for analyses concerning data-model fit, item fit and DIF. As recommended, the sample size was calculated by multiplying the number of items (11/6) by the number of thresholds (3 for polytomous items), with 10–30 persons per threshold [58], indicating that a sample size of 330–990/180–540 (11/6 × 3 × 10/30) can be deemed as adequate for these analyses.

Items with a negative item location estimate could be considered as relatively easy to endorse, whereas the opposite is the case for items with a positive item location estimate. A higher value indicates that the item is having a higher difficulty level and is, consequently, harder to endorse [58].

Convergent and discriminant validity are also facets of construct validity [60]. As the HLS_19_-COM-P intends to measure an aspect of HL, COM-HL, it would be expected that the COM-HL score is positively (moderately) correlated with general HL (GEN-HL) and with navigational HL (HL-NAV; convergent validity) but is still a separate construct (discriminant validity). For convergent validity, Pearson’s correlations were used to assess the associations between COM-HL and GEN-HL and HL-NAV. A positive moderate correlation between these scores would be expected, as the instruments intend to capture different aspects of HL. To assess discriminant validity, the combined procedure of PCA of residuals and paired sample *t*-test was applied to investigate whether the different HL instruments could be considered measuring distinctive constructs. GEN-HL and HL-NAV were measured using the HLS_19_-Q12 and the HLS_19_-NAV instruments, each consisting of 12 items [3].

Rasch analyses were performed using the software RUMM2030Plus [61] and ACER ConQuest 5 [62], the lavaan package [51] for R [63] was applied for CFA, and the correlation analyses were conducted using R.

### 2.5. Missing

On average, there were few missing values. In most countries, the number of missing values varied between 0 and 2 or 3%. In BG data, COM11 had 11% missing values. Conducting Rasch analysis, missing data were handled through full information maximum likelihood estimation (FIML), whereas the other analyses included respondents that had at least 80% of valid responses.

## 3. Results

Table 3 provides details regarding the main characteristics of the samples, including the key demographics, socioeconomic variables, and health status.

### 3.1. Rasch Analyses at the Overall Level

At the overall level, the polytomous scored HLS_19_-COM-P-Q11 displayed misfit in all countries when a sample size of *n* = 660 (20 persons for each of the 33 thresholds (11 items × 3 thresholds)) was considered (Table 4, [39]). Reducing the sample size to 330 in each country, the HLS_19_-COM-P-Q11 displayed acceptable overall data-model fit. Applying a sample size of *n* = 360, the polytomous scored HLS_19_-COM-P-Q6 displayed acceptable overall data-model fit in AT and DE data. Reducing the sample size to 180 (6 items × 3 thresholds × 10) the short version also displayed acceptable data-model fit in the other countries. The proportion of significant different person location estimates across subtests for the HLS_19_-COM-P-Q11 varied between 4.8% (SI; CAPI) and 7.9% (DE; PAPI), and between 3.0% (HU; CATI and SI; CAPI) and 7.5% (DK; CAWI) for HLS_19_-COM-P-Q6, indicating that the scales could be considered sufficiently unidimensional in all countries. The targeting of both the long and the short version could have been better, as the items, on average, were quite easy to endorse (mean person location varying between 1.38 (DE; PAPI) and 2.73 (SI; CAWI), and 1.21 (DE; PAPI) and 2.47 (SI; CAWI) for HLS_19_-COM-P-Q11 and HLS_19_-COM-P-Q6, respectively (Table 4, [39]).

**Figure 3:**
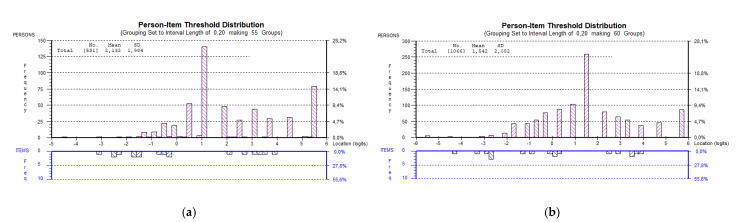
(**a**) Person item distribution for HLS_19_-COM-P-Q6 in the Czech Republic based on CATI data. (**b**) Person item distribution for HLS_19_-COM-P-Q6 in the Czech Republic based on CAWI data.

In countries applying different modes of data collection, higher mean person location was observed for data obtained from CAWI than from CAPI (SI (long and short version) and BG). A higher mean person location was also observed in data obtained from CATI compared with CAWI (CZ; Figure 3a,b).

Assessing fit to the Rasch model based on dichotomized items indicated low power of analysis of fit and a decreased PSI. The results based on dichotomous data also led to a sharp increase in the number of records with extreme scores (4–12 times more extreme records). Reasonable power of analyses of fit was observed only for the DE version of the dichotomized HLS_19_-COM-P-Q11. In DE data, the number of extreme records increased from 81 when analyses were based on polytomous data to 642 when based on dichotomous data. The PSI decreased from 0.89 to 0.62. Hence, the following results from Rasch analyses provided in this paper are based on polytomous data.

### 3.2. Confirmatory Factor Analysis

Regardless of considering polytomous or dichotomous data, most goodness-of-fit indices for both the HLS_19_-COM-P-Q11 and the HLS_19_-COM-P-Q6 could be considered as acceptable when using a one-factor model (Table 5). In DE and SI data, the RMSEA for HLS_19_-COM-P-Q11 was above the recommended reference value. In countries applying different data collection modes, the goodness-of-fit indices were approximately the same, except for the SI HLS_19_-COM-P-Q6 data, where data collected using CAPI had somewhat better fit than CAWI data. Comparing goodness of fit indices based on dichotomous versus polytomous data, the SRMR was either equivalent or lower when analyses were based on polytomous data, whereas the opposite was the case considering RMSEA.

### 3.3. Reliability

Both the HLS_19_-COM-P-Q11 and HLS_19_-COM-P-Q6 obtained acceptable to high reliability indices (Table 6). Based on polytomous data, the PSI, Cronbach’s alpha, and omega for the HLS_19_-COM-P-Q11 varied from 0.86 (AT) to 0.89 (DE), from 0.91 (AT) to 0.94 (SI, CAPI and CAWI), and 0.92 (AT) to 0.95 (SI, CAPI and CAWI), respectively. PSI, Cronbach’s alpha, and omega for the HLS_19_-COM-P-Q6 based on polytomous data varied from 0.75 (AT) to 0.83 (CZ, CATI and CAWI, DK and FR), 0.84 (DE) to 0.90 (BE, DK, SI, CAPI) and from 0.85 (DE) to 0.90 (BE), respectively. A decrease in Cronbach’s alpha and omega was observed when analyses were based on dichotomous data. Both the HLS_19_-COM-P-Q11 and HLS_19_-COM-P-Q6 obtained acceptable values of AVE.

### 3.4. Fit at the Item Level

Using a sample of 990 from each country, the HLS_19_-COM-P-Q11 item COM1 (“describe to your doctor your reasons for coming to the consultation”) displayed significant misfit (*p* < 0.001) in all three countries but had acceptable infit and fit residual. This was also the case for DE (PAPI) and SI (CAPI and CAWI) when reducing the sample size to 660. The item misfit was not significant at Bonferroni 5% for AT considering a sample size of 660. In data from AT (CATI), items COM4 (“get enough time in the consultation with your doctor”; fit residual of 3.87 and infit of 1.21) and COM7 (“understand the words used by your doctor”; fit residual of 3.21 and infit of 1.18) tend to under-discriminate (Table S3: Item fit statistics for HLS_19_-COM-P-Q11 for each country, [39]). The other items displayed acceptable fit.

Most HLS_19_-COM-P-Q11 items worked invariantly across different levels of person factors. However, in DE data, item COM7 (“understand the words used by your doctor”) displayed significant DIF for education, where those having maximum upper secondary school as the highest completed education (ISCED 0 to 3) scored significantly lower than those having higher education despite the same location on the latent trait. This was also evident in SI CAWI data. In addition, item COM7 (“understand the words used by your doctor”) did also display significant DIF for paying bills in SI CAWI data. In SI CAWI data, item COM6 (“get the information you need from your doctor”) displayed DIF for age and education. However, the DIF was not evident when reducing the sample size to 660. The same was the case for age in item COM4 (“get enough time in the consultation with your doctor”) in AT data. In SI CAPI data, item COM10 (“recall the information you get from your doctor”) displayed DIF for age depending on how the variable was categorized (Table S3: Item fit statistics for HLS_19_-COM-P-Q11 for each country, [39]). None of the items displayed DIF when it comes to self-reported health.

Response dependency was observed between items COM1 (“describe to your doctor your reasons for coming to the consultation”) and COM3 (“explain your health concerns to your doctor”) (r = 0.35) in the DE data (not reported in the Table). The response categories worked well for all items in all countries. Applying the HLS_19_-COM-P-Q11, item COM1 (“describe to your doctor your reasons for coming to the consultation”) was the easiest to endorse in all countries, whereas items COM4 (“get enough time in the consultation with your doctor”), COM7 (“understand the words used by your doctor”), COM5 (“express your personal views and preferences to your doctor”) and COM9 (“be involved in decisions about your health in dialogue with your doctor”) were the hardest in AT, DE, SI CAWI and Slovenian CAPI data, respectively (Table S3: Item fit statistics for HLS_19_-COM-P-Q11 for each country, [39]).

For the HLS_19_-COM-P-Q6, most items worked well in most countries. However, item COM4 (“get enough time in the consultation with your doctor”) under-discriminated in BG (CAPI data: fit residual of 3.02 and infit of 1.36, CAWI data: fit residual of 2.01 and infit of 1.27) and DK data (fit residual of 7.21 and infit of 1.36), while item COM10 (“recall the information you get from your doctor”) tend to under-discriminate in BE (fit residual of 3.02 and infit of 1.29), CZ (CATI data: fit residual of 1.98 and infit of 1.39, CAWI data: fit residual of 3.48 and infit of 1.34), DK (fit residual of 4.87 and infit of 1.37) and HU (fit residual of 1.58 and infit of 1.33) data (Table S4: Item fit statistics for HLS_19_-COM-P-Q6 for each country, [39]). The item COM4 (“get enough time in the consultation with your doctor”) also displayed significant DIF across age categories (depending on categorization), level of education and employment status in BG CAPI data. In addition, the item displayed DIF for self-perceived social level in society and self-reported general health status, but this was not significant when reducing the sample size to 360 (Table S4: Item fit statistics for HLS_19_-COM-P-Q6 for each country, [39]). Significant uniform and nonuniform DIF across age categories (depending on categorization) was also observed for item COM3 (“explain your health concerns to your doctor”) in BG CAWI data. For the other countries, no significant DIF was observed when considering a sample size of 360.

No response dependency nor unordered response categories were observed for HLS_19_-COM-P-Q6. In most countries, item COM3 (“explain your health concerns to your doctor”) was the easiest to endorse (in BE data and SI CAWI data, COM8 (“ask your doctor questions in the consultation”) was the easiest and, in HU data, COM10 (“recall the information you get from your doctor”) was the easiest), whereas item COM4 (“get enough time in the consultation with your doctor”) was the hardest in most countries (except for CZ CATI data, CZ CAWI data, FR and SI CAPI data, where COM10 (“recall the information you get from your doctor”), COM9 (“be involved in decisions about your health in dialogue with your doctor”), COM5 (“express your personal views and preferences to your doctor”) and COM9 (“be involved in decisions about your health in dialogue with your doctor”) were the hardest to endorse, respectively; Table S4: Item fit statistics for HLS_19_-COM-P-Q6 for each country).

### 3.5. Invariance across Modes and Countries

**Figure 4:**
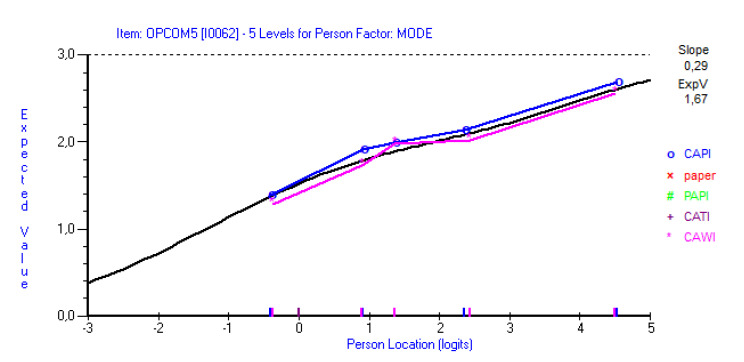
Graphical comparison between means of CAPI and CAWI in Slovenian data for item COM5 (“express your personal views and preferences to your doctor”).

In SI data, the HLS_19_-COM-P-Q11 items COM4 (“get enough time in the consultation with your doctor”), COM5 (“express your personal views and preferences to your doctor”; CAPI > CAWI) and COM7 (“understand the words used by your doctor”; CAPI < CAWI) displayed DIF across mode (*n* = 990). When the sample size was reduced to *n* = 660, DIF was only evident in item COM5 (F-ratio: 12.75, *p* < 0.001; Figure 4). Item COM5 also displayed DIF across mode in data from the SI six-items version, but this was not significant when the sample size was reduced to 540. Using approximately equal sample sizes from CZ CATI and CAWI HLS_19_-COM-P-Q6 data, item COM10 (“recall the information you get from your doctor”) displayed DIF across modes. Item COM3 (“explain your health concerns to your doctor”) did display DIF across mode in BG data. These DIFs were not significant at Bonferroni adjusted 5% level when applying a sample size of 540.

Using equal sample sizes (*n* = 500) from countries applying CATI (AT, CZ, and HU), items COM3 (“explain your health concerns to your doctor”), COM4 (“get enough time in the consultation with your doctor”) and COM10 (“recall the information you get from your doctor”) displayed DIF across the countries. The same items displayed DIF when drawing a random sample of 400 for each country applying CAPI/PAPI (BG, DE, and SI). Using random samples of 500 from countries applying CAWI (BE, BG, CZ, DK, FR and SI), items COM3 (“explain your health concerns to your doctor”), COM4 (“get enough time in the consultation with your doctor”), COM9 (“be involved in decisions about your health in dialogue with your doctor”) and COM10 (“recall the information you get from your doctor”) displayed DIF. The mean person location for the random samples of HLS_19_-COM-P-Q6 data collected using CATI, CAPI/PAPI and CAWI were 2.08, 1.60 and 1.89, respectively.

### 3.6. Convergent and Discriminant Validity

The long and the short version of the instrument were, as expected, highly correlated; r varied from 0.97 (AT) to 0.98 (SI).

The scores obtained from HLS_19_-COM-P-Q11 and the HLS_19-_COM-P-Q6 were moderately to highly correlated with scores of GEN-HL (measured using HLS_19_-Q12) and HL-NAV (measured using HLS_19_-NAV) when analyses were based on polytomous data (Table 7). When conducting analyses based on dichotomous data, the correlation between COM-HL and related HL scores could be considered as small to large (Table 7). Lower correlation coefficients were observed in all countries when analyses were based on dichotomous data compared to polytomous.

Applying the combined PCA of residuals and paired sample *t*-test procedure, the HLS_19_-Q12 (GEN-HL) and HLS_19_-COM-P-Q11/Q6 item residuals loaded on separate components in all countries. The same was the case for the HLS_19_-COM-P-Q11/Q6 and HLS_19_-NAV items. The proportions of significant *t*-tests varied from 21.8% (DE) to 27.7% (SI, CAWI) for HLS_19_-Q12 and HLS_19_-COM-P-Q11, and between 15.1% (CZ, CATI) and 21.9% (BE) for HLS_19_-Q12 and HLS_19_-COM-P-Q6. The proportion of significant *t*-tests between HLS_19_-COM-P-Q11 and HLS_19_-NAV varied between 27.4% (DE) and 36.9% (SI, CAWI), and for HLS19-COM-Q6 and HLS_19_-NAV between 18.5% (AT) and 33.2% (BE).

### 3.7. Distribution of COM-HL Score

The score based on dichotomous items shows a left-skewed distribution with a clear ceiling effect in all countries, for both the long and short versions, regardless of the survey method (see Figure S1a: Distribution of HLS_19_-COM-P-Q11 dichotomous score by country and survey mode; and Figure S1b: Distribution of HLS_19_-COM-P-Q6 dichotomous score by country and survey mode). The score based on polytomous items is rather normally distributed in most countries, both for the long and short version, although, in some countries, the positive extreme values are disproportionately represented in the distribution (Figure S2a: Distribution of HLS_19_-COM-P-Q11 polytomous score by country and survey mode; and Figure S2b: Distribution of HLS_19_-COM-P-Q6 polytomous score by country and survey mode).

## 4. Discussion

Based on our theoretical framework that integrates the idea of COM-HL of Nutbeam [22], the basic competencies of information processing according to the HL framework of the HLS Consortium [3,25,26] and the main communicative tasks of the C-CG framework [32] we succeeded in developing a brief international instrument with acceptable psychometric properties and strong reliability for measuring COM-HL in patient–physician interaction.

### 4.1. Construct Validity and Reliability

The HLS_19_-COM-P-Q11 and HLS_19_-COM-P-Q6 data display acceptable fit to the unidimensional Rasch model (considering a reduced sample size) and acceptable goodness-of-fit indices in CFA. Both HLS_19_-COM-P-Q11 and HLS_19_-COM-P-Q6 gave sufficient unidimensional data, implying that it could be statistically defensible to calculate a total score [66] of COM-HL based on these instruments. Sufficiently high reliability indices do also allow for drawing conclusions about COM-HL both at group and individual levels [40].

However, the targeting of both HLS_19_-COM-P-Q11 and HLS_19_-COM-P-Q6 could have been better. Overall, the items were quite easy to endorse, implying a ceiling effect. Mistargeting might bring decreased reliability, as the precision of the instrument becomes poorer [56]. Hence, the instrument could benefit from adding items that are harder to endorse. On the other hand, for identifying groups with difficulties in HL-COM, the instrument performs well.

Most items displayed acceptable fit to the Rasch model. However, item COM10 (“recall the information you get from your doctor”) under-discriminated in CZ, DK, and HU HLS_19_-COM-P-Q6 data. Under-discriminating items tend also to measure something else that is not positively correlated with the latent trait [67], here, COM-HL. The item is about recalling health information, which might be dependent on other cognitive processes than HL. Hence, in future studies, one might consider replacing this item with item COM11 in the short version of the instrument. This item might also be more in line with the cognitive domain to “apply” health information in the conceptual model of Sørensen et al. [25]. In addition, item COM4 (“get enough time in the consultation with your doctor”) under-discriminated in BG (CAPI) and DK HLS_19_-COM-P-Q6 data. In AT data, the fit residual was also somewhat elevated, but the infit could be deemed as acceptable. Experiences of having sufficient time in consultation with a physician may depend on other things in addition to COM-HL, such as the number and type of health issues that the patient would like to discuss and the patients’ age [68]. In our conceptual framework, there are no other items covering this dimension. However, item COM4 might be replaced by item COM6 (“get the information you need from your doctor”), which could also be an indicator for understanding and following the agenda. In the short version, there are no items covering the dimension opening the session and giving initial information. However, these communicative tasks might be somewhat overlapping, which was confirmed in DE data, as response dependency was observed between items COM1 and COM3.

Few items showed DIF across different levels of person factors and, where DIF was present, there was no consistent pattern across countries. This indicates that the instrument works quite invariantly. However, the HLS_19_-COM-P-Q11 item COM7 (“understand words used by your doctor”) displayed DIF for education in DE and SI CAWI data. The source of DIF might be that patients with low education are less familiar with medical jargon than those with higher education. In BG HLS_19_-COM-P-Q6 CAPI data, COM4 (“get enough time in the consultation with your doctor”) displayed DIF for several person factors. As mentioned above, there might be several reasons that some patients perceive a need for more time in consultation with physicians.

On one hand, the COM-HL score was moderate to highly correlated with GEN-HL and HL-NAV scores, indicating that the instruments measure something common and, consequently, ensure convergent validity. The scores are all based on related instruments, all intending to measure certain aspects of HL. On the other hand, the combined PCA of residuals and *t*-test procedure show that they are measuring distinctive constructs (discriminant validity). Hence, we conclude that the instruments for measuring COM-HL, GEN-HL and HL-NAV are measuring different aspects but could be considered parts of the family of HL instruments. Content and face validity are also ensured by using the theory-based model and definition of communicative HL with physicians in healthcare for selecting and operationalizing the included indicators. Concurrent predictive validity should be further explored in future studies.

### 4.2. Using Dichotomous or Polytomous Scores

Due to a change in the labelling of the response categories from the HLS-EU [26] to the HLS_19_ survey, the HLS_19_ Consortium decided to use dichotomized scores when reporting on HL in the international report [3] to ease the comparison between the surveys. However, no items of the HLS_19_-COM-P-Q11 or HLS_19_-COM-P-Q6 displayed unordered response categories, indicating that the four-point response categories used in the HLS_19_-survey worked well at least for the HLS_19_-COM-P instruments. Conducting Rasch analyses based on dichotomous items did result in an increased number of extreme records. The Cronbach’s alpha values based on polytomous data were also higher than those reported based on dichotomized data, which would also be expected as more response categories yield more scoring points. The correlation between COM-HL and other HL scores was also stronger when analyses were based on polytomous scores. This is in line with Jiao et al. [69] who also found that results based on polytomous scores have slightly higher measurement precision compared to results from dichotomous scoring. Dichotomized scoring might be easier to understand but yields a loss of information and a loss of power [70]. However, dichotomization might reduce the effect of outliers [71].

### 4.3. Data Collection Mode

Different data collection modes were applied across countries and, for some countries, also within the country. The advantage is that the instrument was evaluated for different modes. However, according to Bowling [72], using different modes might bring more response bias than within a single mode. For countries that have used multiple modes, we also found that the mean person location differed across modes. Especially in CZ data, the difference in mean person location estimates between CATI and CAWI data was significant. In CZ, there was a predominance of younger people who responded to the CAWI version, whereas the CATI responses were dominated by older people, as these were hard to reach in CAWI sampling. Hence, the CAWI and CATI samples in CZ are also incomparable due to including different age groups. This was the case also in SI data. Some items did also display DIF across different data collection modes, implying that people responding to questionnaires operationalized by different data collection modes might interpret the items differently. However, the DIF across modes was marginal.

Even though the results should be interpreted with caution, due to DIF across countries, the mean person location was, on average, highest for countries that collected data by CATI and lowest in countries that used CAPI/PAPI. Both might be affected by response bias, but CAPI/PAPI brings less cognitive burden and is usually the most preferred data collection mode for the respondents [72]. Braekman et al. [73] did also find that, in a health survey, responses collected via self-administered modes (web versus PAPI) were more comparable than responses collected via self- and interviewer-administrated modes (web versus CAPI). However, the authors also found that simple and factual questions (such as healthcare use) are less prone to mode differences when comparing self- and interviewer-administered modes. Future studies which intend to compare scores across countries and modes of data collection should take actions to minimize mode effects, such as providing instructions for different modes in order to provide the same perceived stimuli to respondents. To reduce DIF across languages or countries, the translated versions of the instrument should also be assessed to ensure that items are interpreted in the same way across countries.

### 4.4. How to Use the Instrument

The HLS_19_-COM-P intends to measure COM-HL in general adult populations and comprise skills that are necessary to actively participate in an interaction with physicians within a healthcare setting. The COM-HL score is standardized in the range of 0 to 100. Scores are only computed for respondents who have answered at least 80% of the HLS_19_-COM-P items. If less than 80% of the items contain valid responses, the score is set to “missing”. A higher score value signifies a higher level of COM-HL. The score should be interpreted in light of contextual factors related to the health system of the present country.

The instrument belongs to the HLS_19_ Consortium. The use of the instrument is free, but any use of it needs a contractual agreement between the nonprofit applicant and the HLS_19_ Consortium. Further information can be found here: https://m-pohl.net/tools (accessed on 1 June 2022).

### 4.5. Strengths and Limitations

The psychometric properties of the instrument were assessed in large country representative samples from nine countries and are assessed in different data collection modes. The development of the instrument relies on a theory-based definition and conceptual framework of COM-HL.

A limitation is that the instrument only measures patients’ COM-HL in interaction with physicians. However, other healthcare professionals, such as nurses, are also providing health communication in healthcare settings. Hence, a version of the instrument comprising COM-HL in interacting with nurses should also be piloted. As the instrument intends to measure COM-HL in interaction with physicians in a healthcare setting, the instrument should be further tested among patients in clinical settings, especially with relevant indicators for predictive validity of the instrument. The validity and reliability of the HLS_19_-COM-P should also be further explored in people with chronic illnesses.

In most countries, data were collected during the COVID-19 pandemic, which could have had an impact on the responses, as face-to-face encounters in this period were restricted to some extent. However, in DE, data were collected before the pandemic, and the psychometric properties of the HLS_19_-COM-P do not differ much from DE to the other countries.

As the analyses are based on self-reported data, and some also from interviews, there might be a risk of response bias, such as social desirability. There is also a risk of recall misclassification as the experiences of physician–patient interactions might vary because of diverse factors, such as time since the last interaction, frequency of interactions, individual dependence on healthcare, cognitive skills, etc. Selection bias might also have occurred.

## 5. Conclusions

The HLS_19_-COM-P-Q11 and the short version HLS_19_-COM-P-Q6 worked quite well in the nine countries and across different data collection modes, even though misfit was found in a few items in some countries. The scale was also well accepted in all countries, with few missing values. Hence, this instrument could be used for identifying COM-HL in populations, and results from this instrument can be used to give recommendations for policy, practice and for COM-HL interventions (e.g., communication training for physicians). To our understanding, this is the first instrument to measure COM-HL as a separate construct in the family of related HLS instruments. However, the HLS_19_-COM-P should be further evaluated in clinical settings and should be adapted to measure COM-HL also in relation to other health professions.

## Figures and Tables

**Table 1:** Overview of items included in the instrument for measuring communicative health literacy. Items constituting the short version are in italics.

Patients’ Communicative Tasks	Items	
	On a Scale from Very Easy to Very Difficult, How Easy Would You Say It Is for You …	
1. Opening the session and giving initial information	COM1	… to describe to your doctor your reasons for coming to the consultation?
2. Giving full information	COM2	… to make your doctor listen to you without being interrupted?
	COM3	*… to explain your health concerns to your doctor?*
3. Understanding and following the agenda	COM4	*… to get enough time in the consultation with your doctor?*
4. Expressing one’s own views and trusting	COM5	*... to express your personal views and preferences to your doctor?*
5. Understanding and decision making	COM6	… to get the information you need from your doctor?
	COM7	… to understand the words used by your doctor?
	COM8	*… to ask your doctor questions in the consultation?*
	COM9	*... to be involved in decisions about your health in dialogue with your doctor?*
6. Final understanding and agreement	COM10	*… to recall the information you get from your doctor?*
	COM11	… to use the information from your doctor to take care of your health?

**Table 2:** Main characteristics of data collection in countries measuring communicative health literacy.

Country	Item Set	Language	Mode of Data Collection	Sampling Procedure	Period of Data Collection	Number of Respondents ^i^
AT	Q11	German	CATI	Multi-stage random sampling	16 March–26 May 2020	2954
BE	Q6	Dutch, French	CAWI	Quota sampling	30 January–28 February 2020; 1–26 October 2020	1000
BG	Q6	Bulgarian	CAPI, CAWI	Proportional stratified sampling (CAPI) and random quota sampling (CAWI)	15 August–30 November 2020 (CAPI); 1 April–1 June 2021 (CAWI)	859
CZ	Q6	Czech	CATI, CAWI	Random digital procedure (CATI) and random quota sampling (CAWI)	10–24 November 2020	1597
DE	Q11	German	PAPI	Multi-stage random and quota sampling combined	13 December 2019–27 January 2020	2133
DK	Q6	Danish	CAWI	Multi-stage random sampling	11 December 2020–5 February 2021	3600
FR	Q6	French	CAWI	Quota sampling	27 May–5 June 2020; 8–18 January 2021	2003
HU	Q6	Hungarian	CATI	Multi-stage random sampling	2–20 December 2020	1186
SI	Q11	Slovenian	CAPI, self-administered paper and pencil ^ii^, CAWI	Multi-stage random sampling	9–15 March 2020; 9 June 2020–10 August 2020	3342

AT = Austria; BE = Belgium; BG = Bulgaria; CAPI = computer-assisted personal interviews; CATI = computer-assisted telephone interviews; CAWI = computer-assisted web interviews; CZ = Czech Republic; DE = Germany; DK = Denmark; FR = France; HU = Hungary; PAPI = paper-assisted personal interviews; SI = Slovenia. ^i^ The number of respondents who have answered one or more HLS_19_-COM-P items. ^ii^ Only 12 individuals responded using paper and pencil. These records were excluded from the analyses.

**Table 3:** Sample characteristics (in percentages) for the participating countries in HLS_19_, measuring HLS_19_-COM-P, divided by data collection mode.

Characteristic			AT	BE	BG CAPI	BG CAWI	CZ CATI	CZ CAWI	DE	DK	FR	HU	SI CAPI	SI CAWI
*n*			2954	1000	402	457	531	1066	2133	3600	2003	1186	1855	1487
Gender		male	44.2	49.6	29.6	24.5	40.7	51.3	49.6	43.9	49.2	47.8	47.0	45.5
		female	55.8	50.4	70.4	75.5	59.3	48.7	50.2	56.1	50.8	52.2	53.0	54.5
		missing	0.0	0.0	0.0	0.0	0.0	0.0	0.2	0.0	0.0	0.0	0.0	0.0
Age ^#^	Dichotomized	≤45	34.1	43.9	60.5	66.5	18.6	57.0	38.3	21.7	48.6	33.6	28.4	51.8
		≥46	65.8	56.1	37.8	33.5	81.4	43.0	60.8	78.3	51.4	66.4	71.6	48.2
		missing	0.1	0.0	1.7	0.0	0.0	0.0	0.9	0.0	0.0	0.0	0.0	0.0
	Categorized (1st version)	18 to 25 years	6.8	9.0	18.9	20.5	1.7	12.7	9.4	4.4	12.0	7.2	5.3	11.5
		26 to 65 years	70.4	74.7	73.2	75.3	49.7	76.3	65.2	60.7	74.8	65.4	61.9	75.6
		66 years and older	22.7	16.3	6.2	4.2	48.6	11.0	24.5	34.9	13.2	27.4	32.8	12.9
		missing	0.1	0.0	1.7	0.0	0.0	0.0	0.9	0.0	0.0	0.0	0.0	0.0
	Categorized (2nd version)	18 to 45 years	34.1	43.9	60.5	66.5	18.6	57.0	38.3	21.7	48.6	33.6	28.4	51.8
		46 to 75 years	56.3	53.3	37.1	32.4	68.0	41.4	50.0	69.1	51.4	57.5	57.9	44.2
		76 years and older	9.5	2.8	0.7	1.1	13.4	1.6	10.8	9.2	0.0	8.9	13.7	4.0
		missing	0.1	0.0	1.7	0.0	0.0	0.0	0.9	0.0	0.0	0.0	0.0	0.0
Highest level of completed education		Upper secondary school (ISCED 0 to 3)	61.9	14.7	32.3	22.1	86.6	71.8	54.4	15.1	17.9	70.4	80.9	54.3
		above	38.1	84.1	66.2	77.5	13.2	28.2	43.5	84.8	82.1	29.6	19.1	45.7
		missing	0.0	1.2	1.5	0.4	0.2	0.0	2.1	0.1	0.0	0.0	0.0	0.0
Status of employment		employed	59.7	57.7	81.4	87.8	34.5	72.5	60.5	55.2	67.5	56.9	44.8	70.3
		unemployed or retired	40.0	38.4	14.4	9.4	65.2	27.2	38.1	40.9	32.5	42.5	54.9	28.4
		missing	0.3	3.9	4.2	2.8	0.3	0.3	1.4	3.9	0.0	0.6	0.3	1.3
Ability to pay bills		easy	85.8	62.4	61.5	63.0	81.4	67.4	73.7	92.8	74.6	67.4	56.2	61.2
		difficult	13.3	37.6	33.3	35.2	18.3	32.6	22.5	6.9	25.4	31.3	42.5	38.7
		missing	0.9	0.0	5.2	1.8	0.4	0.0	3.8	0.3	0.0	1.3	1.3	0.1
Self-perceived level in society (1 to 10)		level 4 or lower ^i^	6.8	10.6	11.7	9.0	13.9	17.4	17.3	11.4	20.3	26.1	25.9	20.6
		level 5 or higher	87.2	89.4	77.9	79.0	84.0	82.6	80.0	88.3	79.7	72.6	71.1	79.1
		missing	6.0	0.0	10.4	12.0	2.1	0.0	2.7	0.3	0.0	1.3	3.0	0.3
Self-reported general health		good or fair	97.0	92.1	96.3	96.3	85.5	91.5	93.0	92.6	92.6	91.1	90.2	96.3
		bad	2.9	7.9	3.5	3.3	14.3	8.5	6.9	7.3	7.4	8.8	9.7	3.6
		missing	0.1	0.0	0.2	0.4	0.2	0.0	0.1	0.1	0.0	0.1	0.1	0.1

AT = Austria; BE = Belgium; BG = Bulgaria; CAPI = computer-assisted personal interviews; CATI = computer-assisted telephone interviews; CAWI = computer-assisted web interviews; CZ = Czech Republic; DE = Germany; DK = Denmark; FR = France; HU = Hungary; SI = Slovenia. ^#^ We used different categorizations of age when conducting analyses of differential item functioning to explore if the categorization had an impact on the results in these analyses. Patients aged ≥ 76 are perceived as a vulnerable subpopulation. In FR, the sample was collected among people aged below 75. ^i^ People with a score of ≤4 are considered at a low level in society [64,65].

**Table 4:** Overall fit for HLS_19_-COM-P-Q11 (left) and HLS_19_-COM-P-Q6 (right) to the partial credit parametrization of the unidimensional polytomous Rasch model.

	Q11				Q6											
	AT *n* = 2954	DE *n* = 2133	SI *n* = 1856	SI *n* = 1487	AT *n* = 2952	BE *n* = 1000	BG *n* = 402	BG *n* = 457	CZ *n* = 531	CZ *n* = 1066	DE *n* = 2133	DK *n* = 3600	FR *n* = 2003	HU *n* = 1186	SI *n* = 1855	SI *n* = 1487
mode	CATI	PAPI	CAPI	CAWI	CATI	CAWI	CAPI	CAWI	CATI	CAWI	PAPI	CAWI	CAWI	CATI	CAPI	CAWI
χ^2^, *p*	81.5, <0.001	84.5, <0.001	94.2, <0.001	108.1, <0.001	33.2, 0.1	57.3, <0.001	62.9, <0.001	93.2, <0.001	84.4, <0.001	51.3, 0.001	34.6, 0.07	86.7, <0.001	44.5, 0.01	52.1, 0.001	45.8, 0.005	47.7, 0.003
Mean person location	2.57	1.38	2.55	2.73	2.39	2.20	1.34	1.58	2.13	1.54	1.21	1.97	1.85	1.88	2.36	2.47
Dimensionality, % (lower 95% CI proportion)	6.1 (5.3)	7.9 (7.0)	4.8 (3.8)	6.9 (5.8)	5.3 (4.5)	4.4 (3.0)	6.5 (4.4)	5.3 (3.3)	5.8 (4.0)	5.1 (3.8)	5.0 (4.1)	7.5 (6.7)	3.6 (2.6)	3.0 (1.7)	3.0 (2.0)	4.4 (3.3)

AT = Austria; BE = Belgium; BG = Bulgaria; CAPI = computer-assisted personal interviews; CATI = computer-assisted telephone interviews; CAWI = computer-assisted web interviews; CI = confidence interval; CZ = Czech Republic; DE = Germany; DK = Denmark; FR = France; HU = Hungary; PAPI = paper-assisted personal interviews; SI = Slovenia. Chi-square (χ^2^) is based on *n* = 660 and 360 for HLS_19_-COM-P-Q11 and HLS_19_-COM-P-Q6, respectively [39].

**Table 5:** Fit indices for the one-factor model of the HLS_19_-COM-P-Q11 (left) and HLS_19_-COM-P-Q6 (right), for each country, divided by data collection mode. Analyses are based on both polytomous and dichotomous (marked in grey) data.

		Q11				Q6											
Fit-Indices		AT *n* = 2766	DE *n* = 2064	SI *n* = 1781	SI *n* = 1471	AT *n* = 2827	BE *n* = 1000	BG *n* = 333	BG *n* = 394	CZ *n* = 504	CZ *n* = 1066	DE *n* = 2101	DK *n* = 3574	FR *n* = 2003	HU *n* = 1125	SI *n* = 1788	SI *n* = 1477
mode		CATI	PAPI	CAPI	CAWI	CATI	CAWI	CAPI	CAWI	CATI	CAWI	PAPI	CAWI	CAWI	CATI	CAPI	CAWI
SRMR	polytomous data	0.04	0.07	0.04	0.05	0.03	0.02	0.03	0.03	0.02	0.02	0.03	0.01	0.02	0.03	0.01	0.03
	dichotomous data	0.06	0.07	0.06	0.07	0.03	0.04	0.06	0.06	0.07	0.02	0.03	0.02	0.03	0.05	0.02	0.03
RMSEA	polytomous data	0.07	0.11	0.10	0.10	0.06	0.05	0.07	0.07	0.03	0.03	0.07	0.03	0.05	0.07	0.03	0.07
	dichotomous data	0.02	0.05	0.03	0.04	0.00	0.02	0.02	0.04	0.00	0.00	0.03	0.01	0.02	0.02	0.00	0.01
RMSEA; CI, lower	polytomous data	0.06	0.11	0.10	0.09	0.05	0.03	0.04	0.04	0.00	0.01	0.05	0.02	0.04	0.05	0.01	0.05
	dichotomous data	0.02	0.05	0.03	0.03	0.00	0.00	0.00	0.00	0.00	0.00	0.01	0.00	0.00	0.00	0.00	0.00
RMSEA; CI, upper	polytomous data	0.07	0.12	0.11	0.11	0.07	0.07	0.11	0.10	0.06	0.05	0.08	0.04	0.06	0.08	0.04	0.08
	dichotomous data	0.03	0.06	0.04	0.04	0.02	0.05	0.07	0.07	0.05	0.03	0.04	0.02	0.04	0.05	0.01	0.03
RMSEA; *p*-value	polytomous data	0.00	0.00	0.00	0.00	0.06	0.52	0.12	0.18	0.79	0.91	0.02	1.00	0.52	0.06	1.00	0.02
	dichotomous data	1.00	0.16	1.00	1.00	1.00	0.98	0.80	0.71	0.95	1.00	1.00	1.00	1.00	0.98	1.00	1.00
CFI	polytomous data	1.00	0.98	1.00	1.00	1.00	1.00	1.00	1.00	1.00	1.00	1.00	1.00	1.00	1.00	1.00	1.00
	dichotomous data	0.99	0.99	1.00	0.99	1.00	1.00	1.00	1.00	1.00	1.00	1.00	1.00	1.00	1.00	1.00	1.00
TLI	polytomous data	0.99	0.98	1.00	0.99	1.00	1.00	1.00	1.00	1.00	1.00	0.99	1.00	1.00	1.00	1.00	1.00
	dichotomous data	0.99	0.98	0.99	0.99	1.00	1.00	1.00	1.00	1.00	1.00	1.00	1.00	1.00	1.00	1.00	1.00
GFI	polytomous data	1.00	0.99	1.00	1.00	1.00	1.00	1.00	1.00	1.00	1.00	1.00	1.00	1.00	1.00	1.00	1.00
	dichotomous data	1.00	0.99	1.00	0.99	1.00	1.00	1.00	1.00	1.00	1.00	1.00	1.00	1.00	1.00	1.00	1.00
AGFI	polytomous data	0.99	0.98	0.99	0.99	0.99	1.00	0.99	0.99	1.00	1.00	0.99	1.00	1.00	0.99	1.00	0.99
	dichotomous data	0.99	0.98	0.99	0.99	1.00	1.00	0.99	0.99	0.99	1.00	0.99	1.00	1.00	1.00	1.00	1.00

AT = Austria; BE = Belgium; BG = Bulgaria; CAPI = computer-assisted personal interviews; CATI = computer-assisted telephone interviews; CAWI = computer-assisted web interviews; CFI = comparative fit index; CZ = Czech Republic; DE = Germany; DK = Denmark; FR = France; GFI = goodness-of-fit index; HU = Hungary; PAPI = paper-assisted personal interviews; RMSEA = root mean square error of approximation; SI = Slovenia. SRMR = standardized root mean square residual; TLI = Tucker–Lewis index.

**Table 6:** Reliability indices for the HLS_19_-COM-P-Q11 (left) and HLS_19_-COM-P-Q6 (right), for each country, divided by data collection mode. Calculation of Person separation index is based on polytomous data, whereas Cronbach’s alpha, omega and average variance extracted are displayed for both polytomous and dichotomous (marked in grey) data.

		Q11				Q6											
		AT CATI	DE PAPI	SI CAPI	SI CAWI	AT CATI	BE CAWI	BG CAPI	BG CAWI	CZ CATI	CZ CAWI	DE PAPI	DK CAWI	FR CAWI	HU CATI	SI CAPI	SI CAWI
Person separation index	polytomous data ^i^	0.86	0.89	0.88	0.88	0.75	0.82	0.80	0.82	0.83	0.83	0.81	0.83	0.83	0.77	0.78	0.79
	dichotomous data ^ii^	-	-	-	-	-	-	-	-	-	-	-	-	-	-	-	-
Cronbach’s alpha	polytomous data	0.91	0.90	0.94	0.94	0.86	0.90	0.87	0.88	0.87	0.88	0.84	0.90	0.89	0.87	0.90	0.89
	dichotomous data	0.78	0.84	0.87	0.86	0.68	0.80	0.81	0.80	0.70	0.81	0.74	0.78	0.80	0.78	0.80	0.79
Omega	polytomous data	0.92	0.93	0.95	0.95	0.86	0.90	0.87	0.89	0.87	0.88	0.85	0.89	0.89	0.87	0.89	0.89
	dichotomous data	0.80	0.86	0.91	0.90	0.71	0.81	0.84	0.83	0.74	0.82	0.76	0.80	0.81	0.81	0.82	0.81
Average variance extracted	polytomous data	0.63	0.58	0.74	0.71	0.64	0.71	0.67	0.67	0.67	0.66	0.57	0.71	0.70	0.67	0.73	0.70
	dichotomous data	0.55	0.57	0.73	0.68	0.57	0.67	0.72	0.69	0.62	0.67	0.56	0.67	0.67	0.68	0.73	0.70

AT = Austria; BE = Belgium; BG = Bulgaria; CAPI = computer-assisted personal interviews; CATI = computer-assisted telephone interviews; CAWI = computer-assisted web interviews; CZ = Czech Republic; DE = Germany; DK = Denmark; FR = France; HU = Hungary; PAPI = paper-assisted personal interviews; SI = Slovenia. ^i^ [39]; ^ii^ Due to low power, it could not be calculated.

**Table 7:** Correlation between COM-HL scores (based on HLS_19_-COM-P-Q11 to the left and HLS_19_-COM-P-Q6 to the right) and general (GEN-HL) and navigational (HL-NAV) health literacy scores, based on polytomous and dichotomous (marked in grey) data. Results are divided by country and data collection mode.

		Q11				Q6											
		AT *n* = 2954	DE *n* = 2133	SI *n* = 1856	SI *n* = 1487	AT *n* = 2952	BE *n* = 1000	BG *n* = 402	BG *n* = 457	CZ *n* = 531	CZ *n* = 1066	DE *n* = 2133	DK *n* = 3600	FR *n* = 2003	HU *n* = 1186	SI *n* = 1855	SI *n* = 1487
mode		CATI	PAPI	CAPI	CAWI	CATI	CAWI	CAPI	CAWI	CATI	CAWI	PAPI	CAWI	CAWI	CATI	CAPI	CAWI
GEN-HL	polytomous data	0.54	0.59	0.59	0.53	0.52	0.35	0.56	0.65	0.49	0.49	0.56	0.55	0.60	0.47	0.58	0.51
	dichotomous data	0.37	0.54	0.51	0.44	0.34	0.27	0.46	0.53	0.39	0.45	0.50	0.47	0.52	0.36	0.49	0.41
HL-NAV	polytomous data	0.57	0.55	0.55	0.53	0.56	0.44	-	-	0.44	0.49	0.54	-	0.51	-	0.54	0.52
	dichotomous data	0.49	0.48	0.48	0.42	0.46	0.36	-	-	0.38	0.45	0.45	-	0.44	-	0.48	0.41

AT = Austria; BE = Belgium; BG = Bulgaria; CAPI = computer-assisted personal interviews; CATI = computer-assisted telephone interviews; CAWI = computer-assisted web interviews; CZ = Czech Republic; DE = Germany; DK = Denmark; FR = France; Gen-HL = general health literacy; HL-NAV = navigational health literacy; HU = Hungary; PAPI = paper-assisted personal interviews; SI = Slovenia. PAPI: paper-assisted personal interviews. BG, DK, and HU did not measure HL-NAV.

## Data Availability

Information about data supporting reported results can be found on the M-POHL webpage, https://m-pohl.net/Design_Methods (accessed on 1 June 2022).

## Supplementary Materials

The following supporting information can be downloaded at: www.mdpi.com/article/10.3390/ijerph191811592/s1, Figure S1a. Distribution of HLS_19_-COM-P-Q11 dichotomous score by country and survey mode, Figure S1b. Distribution of HLS_19_-COM-P-Q6 dichotomous score by country and survey mode, Figure S2a. Distribution of HLS_19_-COM-P-Q11 polytomous score by country and survey mode, Figure S2b. Distribution of HLS_19_-COM-P-Q6 polytomous score by country and survey mode, Table S1: Fit indices considered in confirmatory factor analysis, Table S2: Analyses and tests with reference values considered in Rasch analyses, Table S3: Item fit statistics for HLS_19_-COM-P-Q11 for each country, Table S4: Item fit statistics for HLS_19_-COM-P-Q6 for each country.
