# Supplementary material for: The HLS19-COM-P, a New Instrument for Measuring Communicative Health Literacy in Interaction with Physicians: Development and Validation in Nine European Countries
Source: Int J Environ Res Public Health. 2022 Sep 14;19(18):11592. doi: 10.3390/ijerph191811592 (PMC9517091; doi:10.3390/ijerph191811592)
Supplement: Supplementary file 1 [file ijerph-19-11592-s001.zip › Supplementary_FigureS1a_FigureS1b.pdf]

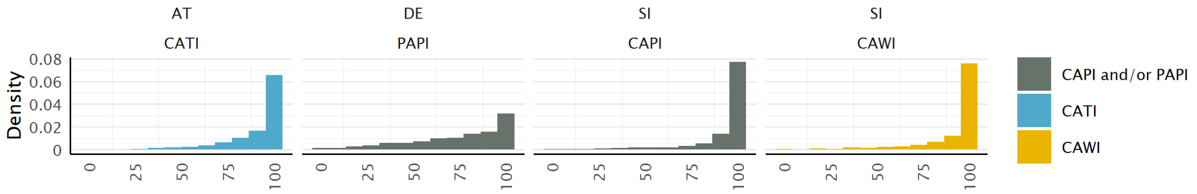

Figure S1a. Distribution of HLS19-COM-P-Q11 dichotomous score by country and survey mode. CAPI= computer personal interviews; CATI computer-assisted telephone interviews; CAWI= computer-assisted web interviews; PAPI= paper-assisted personal interviews.

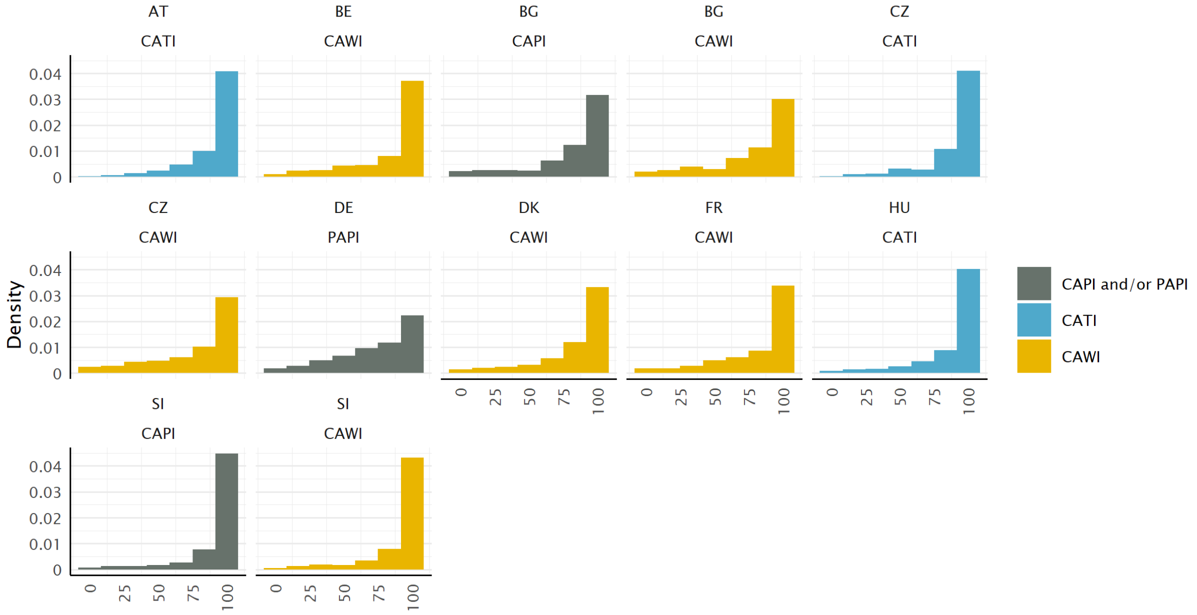

Figure S1b. Distribution of HLS19-COM-P-Q6 dichotomous score by country and survey mode. CAPI= computer personal interviews; CATI computer-assisted telephone interviews; CAWI= computer-assisted web interviews; PAPI= paper-assisted personal interviews.
