# Supplementary material for: The HLS19-COM-P, a New Instrument for Measuring Communicative Health Literacy in Interaction with Physicians: Development and Validation in Nine European Countries
Source: Int J Environ Res Public Health. 2022 Sep 14;19(18):11592. doi: 10.3390/ijerph191811592 (PMC9517091; doi:10.3390/ijerph191811592)
Supplement: Supplementary file 1 [file ijerph-19-11592-s001.zip › Supplementary_TableS3_Item fitHLS19-COM-P-Q11.pdf]

Table S3. Item fit statistics for HLS_{19}-COM-P-Q11 for each country

Country	Item	Location	Fit.res.[i]	Chi sq *n*=990 (df=4)[i]	Chi sq prob (df=4)[i]	Infit MNSQ[i]	DIF[i] *n*=990
**Austria**	COM1	-1.105	1.140	20.956	<0.001*	1.07	-
	COM2	0.100	-1.807	1.936	0.748	1.02	-
	COM3	-0.584	-4.478	9.510	0.050	0.95	-
	COM4	1.204	3.868	19.668	0.001	1.21	age (dichotomized), age (categorized 2nd version)
	COM5	0.345	-7.175	9.744	0.045	0.89	-
	COM6	0.165	-6.666	14.962	0.005	0.90	-
	COM7	0.380	3.209	14.114	0.007	1.18	-
	COM8	-0.260	-6.835	13.580	0.009	0.91	-
	COM9	-0.058	-5.135	7.025	0.135	0.92	-
	COM10	-0.132	0.23	6.174	0.187	1.12	-
	COM11	-0.055	-1.219	4.636	0.327	1.04	-
**Germany**	COM1	-1.948	-1.152	31,491	<0.001*	1.05	-
	COM2	-0.081	-2.428	2,474	0.649	1.00	-
	COM3	-1.139	-4.387	21,444	<0.001	0.93	-
	COM4	1.132	2.312	9,542	0,049	1.11	-
	COM5	0.543	-3.771	5,285	0,259	0.96	-
	COM6	0.047	-5.928	8,83	0,066	0.86	-
	COM7	1.163	2.126	12,039	0,017	1.10	education*
	COM8	-0.212	-6.269	11,362	0,023	0.89	-
	COM9	0.444	-2.298	6,072	0,194	0.97	-
	COM10	-0.027	2.169	11,987	0,018	1.15	-
	COM11	0.08	0.820	6,212	0,184	1.11	-
**Slovenia**	COM1	-0.600	-2.120	30.502	<0.001*	1.02	-
**CAWI**	COM2	-0.098	-4.925	7.308	0,121	0.92	-
	COM3	-0.098	-8.006	11.917	0,018	0.83	-
	COM4	0.983	1.124	22.221	<0.001	1.18	-
	COM5	0.768	-3.391	20.394	<0.001	1.01	-

	COM6	0.257	-6.280	12.622	0.013	0.89	age (dichotomized), education
	COM7	-0.376	-2.029	6.824	0.146	1.11	education*, ability to pay bills*
	COM8	-0.466	-4.440	8.925	0.063	1.00	-
	COM9	0.306	-7.518	25.793	<0.001*	0.81	-
	COM10	-0.349	-1.244	11.826	0.019	1.14	-
	COM11	-0.327	-1.260	3.842	0.428	1.09	-
**Slovenia**[x]	COM1	-0.818	-5.025	24.671	<0.001*	1.02	-
**CAPI**	COM2	-0.271	-7.792	2.599	0.458	0.96	-
	COM3	-0.361	-11.284	15.547	0.001	0.84	-
	COM4	0.555	-3.548	16.233	0.001	1.16	-
	COM5	0.131	-10.365	7.585	0.055	0.85	--
	COM6	0.051	-8.980	6.403	0.094	0.89	-
	COM7	0.060	-5.481	1.65	0.648	1.03	-
	COM8	-0.194	-9.367	15.64	0.002	0.88	-
	COM9	0.744	-4.006	45.754	<0.001*	1.08	-
	COM10	0.008	-4.245	3.591	0.309	1.13	age (categorized 2nd version)*
	COM11	0.095	-4.397	2.019	0.568	1.07	-

*statistically significant when sample size = 660; [x]df = 3; CAPI = computer-assisted personal interviews; CAWI = computer-assisted web interviews; Chi sq prob = Chi-square probability; DIF = differential item functioning; Fit.resid. = Fit residual; MNSQ = mean square.
[i] Reference: [39].
