# Supplementary material for: The HLS19-COM-P, a New Instrument for Measuring Communicative Health Literacy in Interaction with Physicians: Development and Validation in Nine European Countries
Source: Int J Environ Res Public Health. 2022 Sep 14;19(18):11592. doi: 10.3390/ijerph191811592 (PMC9517091; doi:10.3390/ijerph191811592)
Supplement: Supplementary file 1 [file ijerph-19-11592-s001.zip › Supplementary_TableS4_ItemfitHLS19-COM-P-Q6.pdf]

Table S4. Item fit statistics for HLS$_{19}$-COM-P-Q6 for each country

Country	Item	Location	Fit.res.[i]	Chi sq [i] *n*=540	Chi sq prob [i]	Infit MNSQ [i]	DIF [i] *n*=540
**Austria**	COM3	-0.663	-0.903	8.088	0.088	1.03	-
	COM4	1.124	3.215	12.101	0.017	1.21	-
	COM5	0.244	-7.609	9.920	0.042	0.88	-
	COM8	-0.371	-5.605	9.087	0.059	0.92	-
	COM9	-0.127	-3.930	3.519	0.475	0.93	-
	COM10	-0.207	2.536	7.092	0.131	1.15	-
**Belgium**[i]	COM3	-0.411	0.276	5.107	0.277	1.07	-
	COM4	0.467	0.903	8.181	0.085	1.16	-
	COM5	0.159	-4.997	16.462	0.002	0.85	-
	COM8	-0.522	-7.954	28.393	<0.001*	0.76	-
	COM9	0.006	-3.357	8.083	0.089	0.91	-
	COM10	0.301	3.020	19.731	<0.001	1.29	-
**Bulgaria**	COM3	-0.905	-2.688	10.653	0.031	0.97	-
**CAPI, n=402**[a]	COM4	0.785	3.016	23.911	<0.001*	1.36	age (categorized 1st version)*, education*, status of employment*, level in society, general health
	COM5	0.031	-4.676	4.155	0.385	0.80	-
	COM8	-0.490	-4.983	13.011	0.011	0.79	age (categorized 1st version)[#]
	COM9	0.013	-4.157	5.329	0.255	0.88	-
	COM10	0.567	0.078	5.844	0.211	1.13	-
**Bulgaria**	COM3	-1.154	0.111	25.797	<0.001*	1.14	age (categorized 1st version)*[#],
**CAWI, n=457**	COM4	1.046	2.008	29.962	<0.001*	1.27	-
	COM5	-0.078	-4.804	9.692	0.046	0.77	-
	COM8	-0.568	-4.853	11.482	0.022	0.82	-
	COM9	0.218	-2.440	7.274	0.122	0.89	-
	COM10	0.535	0.832	8.968	0.062	1.15	-

**Czechia**	COM3	-0.634	-2,271	27,561	<0.001*	0.97	-
**CATI**	COM4	0.378	-1,221	4,117	0,390	1.10	-
	COM5	-0.115	-3,714	14,909	0,005	0.90	-
	COM8	-0.445	-4,305	16,832	0,002	0.84	status of employment
	COM9	0.211	-2,613	9,012	0,061	0.93	-
	COM10	0.605	1,982	32,589	<0.001*	1.39	-
**Czechia**	COM3	-0.983	-2.250	15.824	0.003	0.97	-
**CAWI**	COM4	0.373	0.459	7.813	0.099	1.09	-
	COM5	0.351	-6.171	10.307	0.036	0.85	-
	COM8	-0.429	-5.683	11.631	0.020	0.86	-
	COM9	0.485	-3.571	11.664	0.020	0.91	-
	COM10	0.202	3.482	19.643	0.001	1.34	-
**Denmark**	COM3	-0.781	-6.843	16.221	0.002	0.95	-
	COM4	1.273	7.212	42.432	<0.001*	1.36	-
	COM5	0.011	-15.154	18.448	0.001	0.79	-
	COM8	-0.483	-16.249	18.971	0.001	0.77	-
	COM9	-0.125	-11.622	11.426	0.022	0.85	-
	COM10	0.103	4.871	22.522	<0.001*	1.37	-
**France**[i]	COM3	-0.792	-3.915	16.762	0.002	1.02	-
	COM4	0.567	-0.147	6.531	0.162	1.16	-
	COM5	0.585	-4.252	10.354	0.035	0.98	-
	COM8	-0.678	-7.4	16.791	0.002	0.90	-
	COM9	0.163	-7.955	9.113	0.058	0.85	-
	COM10	0.154	0.572	7.219	0.125	1.21	-
**Germany**	COM3	-1.238	-1.943	16.259	0.003	1.00	-
	COM4	0.997	2.116	9.979	0.041	1.10	-
	COM5	0.407	-5.113	4.385	0.357	0.92	-
	COM8	-0.346	-6.135	8.21	0.084	0.88	-
	COM9	0.324	-2.241	2.808	0.591	0.94	-
	COM10	-0.144	3.745	10.241	0.037	1.17	-

**Hungary**	COM3	-0.266	-4.453	5.619	0.230	1.01	-
	COM4	0.751	-3.242	12.854	0.012	1.08	-
	COM5	0.201	-7.962	11.780	0.019	0.81	-
	COM8	-0.355	-7.449	7.902	0.095	0.86	-
	COM9	0.184	-5.748	7.144	0.129	0.91	-
	COM10	-0.515	1.577	32.830	<0.001*	1.33	-
**Slovenia**	COM3	-0.299	-6.303	7.088	0.131	0.88	-
**CAWI**	COM4	0.795	0.122	11.34	0.022	1.19	-
	COM5	0.550	-4.992	13.948	0.008	0.92	-
	COM8	-0.669	-4.487	8.882	0.064	0.99	-
	COM9	0.128	-6.192	14.208	0.007	0.87	-
	COM10	-0.504	1.022	15.928	0.003	1.24	-
**Slovenia**	COM3	-0.507	-9.109	14.951	0.005	0.91	-
**CAPI**	COM4	0.406	-4.702	5.825	0.213	1.10	-
	COM5	-0.031	-10.789	5.324	0.256	0.83	-
	COM8	-0.348	-8.637	14.947	0.005	0.89	-
	COM9	0.589	-5.509	22.011	<0.001	1.00	-
	COM10	-0.109	-2.679	5.617	0.230	1.19	-

[i] complete data

[#]non-uniform DIF. All other DIFs were uniform.

CAPI= computer-assisted personal interviews; CATI computer-assisted telephone interviews; CAWI= computer-assisted web interviews; Chi sq prob = Chi-square probability; Fit.resid. = Fit residual; MNSQ = mean square; PAPI= paper-assisted personal interviews.

[i] Reference: [39].
