# Supplementary material for: The HLS19-COM-P, a New Instrument for Measuring Communicative Health Literacy in Interaction with Physicians: Development and Validation in Nine European Countries
Source: Int J Environ Res Public Health. 2022 Sep 14;19(18):11592. doi: 10.3390/ijerph191811592 (PMC9517091; doi:10.3390/ijerph191811592)
Supplement: Supplementary file 1 [file ijerph-19-11592-s001.zip › TableS1Supplementary about CFA.pdf]

Table S1. Fit indices considered in confirmatory factor analysis

**Category**	**Fit indices**	**Good-model-fit criteria**[#]
Absolute Fit	Standardized Root Mean Square Residual (SRMR)	Values <0.05 indicate good model fit; Values <0.08 are considered acceptable
	Goodness of Fit Index (GFI)	Values ≥0.95 indicate good model fit Values >0.9 are considered acceptable
	Adjusted Goodness of Fit (AGFI)	Values ≥0.95 indicate good model fit Values >0.9 are considered acceptable
Comparative Fit	Comparative Fit Index (CFI)	Values ≥0.95 indicate good model fit Values >0.9 are considered acceptable
	Tucker-Lewis Index (TLI)	
Parsimonious Fit	Root-mean-squared error of approximation (RMSEA)[i]	Values <0.05 indicate good model fit; Values <0.08 are considered acceptable

[i]Sometimes also grouped as *Absolute fit*
References: [52, 53].
