# Supplementary material for: The HLS19-COM-P, a New Instrument for Measuring Communicative Health Literacy in Interaction with Physicians: Development and Validation in Nine European Countries
Source: Int J Environ Res Public Health. 2022 Sep 14;19(18):11592. doi: 10.3390/ijerph191811592 (PMC9517091; doi:10.3390/ijerph191811592)
Supplement: Supplementary file 1 [file ijerph-19-11592-s001.zip › TableS2Supplementary about Rasch analyses.pdf]

Table S2. Analyses and tests with reference values considered in Rasch analyses

Analyses	Tests	Reference value	Software
Data-model fit	Total item χ^2 with *p*-values	Insignificant total item χ^2 indicates good data-model fit	RUMM2030Plus
Targeting	Person-item location	The distribution of person location estimates is compared to the distribution of the item location estimates. Mean person location values close to 0 indicate good targeting between the person's ability and the item difficulty[a]	RUMM2030Plus
Dimensionality	PCA/*t*-test procedure	Proportions of significant *t*-tests >5 % indicate multidimensionality[b]	RUMM2030Plus
Response dependence	Residual correlations	>0.3 indicates response dependence[c]	RUMM2030Plus
Item fit	χ^2 statistics with *p*-values	*P*-values <5 % Bonferroni-adjusted are deemed as misfit	RUMM2030Plus
	fit residuals	Values below/above -2.5/ 2.5 indicate over-/under-discrimination[a]	RUMM2030Plus
	Infit weighted mean square (MNSQ)	Infit values between 0.7 and 1.3 was considered as sufficient. Values lower than 0.7 indicate over-discrimination, whereas values higher above 1.3 indicate under-discrimination[d]	ConQuest5
Differential item functioning (DIF)	Analysis of variance (two-way ANOVA)	High F-values with probability <5% Bonferroni-adjusted indicates DIF[e]	RUMM2030Plus
Ordering of response categories	Ordering of thresholds	Inspecting the thresholds to determine whether they are in correct order[e]	RUMM2030Plus
Reliability	Person separation index (PSI)	>0.85 at individual level >0.65 at group level[f]	RUMM2030Plus

Suggestions for references (a selection) for the different analyses: [a][45], [b][6-48], [c][55], [d][54], [e][56-58], [f][40].
